# Patient expectations and healthcare professional education in obesity care: A comparative qualitative study

**DOI:** 10.1016/j.obpill.2026.100337

**Published:** 2026-09-28

**Authors:** Tak Ying Louise Ko, Francisca Contreras, Carel W. Le Roux, Werd Al-Najim

**Affiliations:** a Diabetes Complications Research Centre, University College Dublin, Ireland; b Diabetes Research Centre, Ulster University, UK

**Keywords:** Obesity management, Patient expectations, Weight stigma, Qualitative research, Medical education

## Abstract

**Background:**

Patient expectations of obesity care and healthcare professionals’ (HCPs) educational preparation have largely been studied independently. Comparing these perspectives may reveal whether university education prepares graduates to translate obesity-related knowledge into the capabilities patients consider important in clinical care.

**Methods:**

This was a secondary comparative qualitative analysis conducted using previously collected interviews with 20 adults living with obesity and 21 newly qualified HCPs. Original transcripts were re-analysed in relation to a new comparative research question. Patterns within each dataset were examined and then synthesised across datasets to identify how patient-reported expectations corresponded with HCP-reported university preparation.

**Results:**

The comparison revealed that apparent alignment in broad principles often diverged at the level of clinical application. Both cohorts recognised obesity as multifactorial and emphasised sensitive communication and appropriate management; however, patients expected these principles to shape how obesity was understood, discussed and acted upon within their individual circumstances. HCPs described variable preparation for translating conceptual knowledge into individualised clinical encounters, weight conversations, treatment-oriented discussions and navigation of onward care. A further cross-dataset pattern concerned integration: patients experienced obesity care as fragmented across conditions and specialties, while HCPs described learning about obesity across disconnected subjects and clinical contexts. Across both cohorts, the main divergence was in how obesity-related knowledge was integrated and applied in clinical practice.

**Conclusions:**

The comparative synthesis suggests that the greatest divergence between patient expectations and HCP education lies primarily in translating fragmented obesity-related learning into coherent clinical practice. University education may therefore benefit from greater integration of obesity-specific knowledge, communication and care pathways, rather than solely increasing curricular content.

## Graphical abstract

**Figure:**
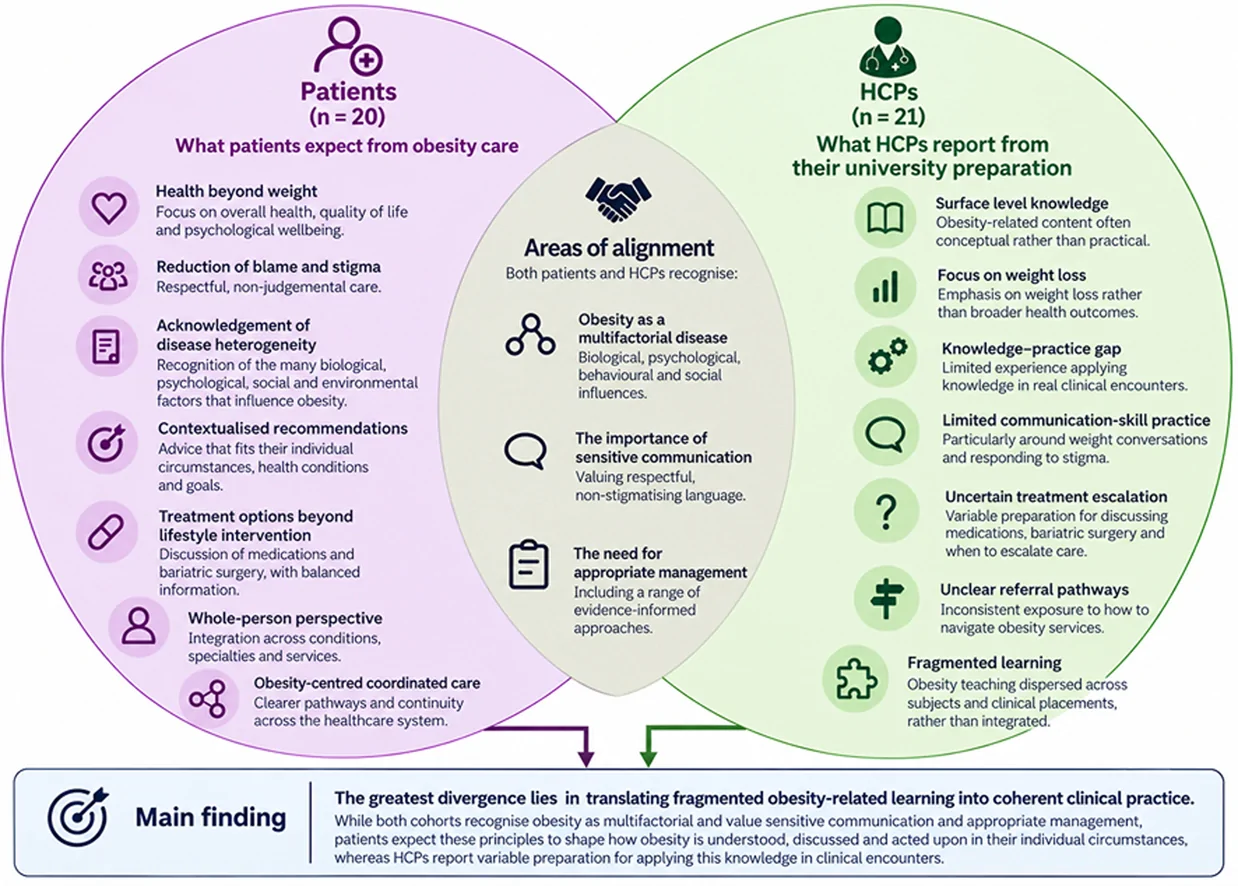

## 1 Introduction

Advances in obesity science have changed the context in which obesity care is delivered, with greater recognition of obesity as a complex chronic disease and an expanding range of management approaches. Alongside these developments, people living with obesity report expectations for care that is evidence-informed, individualised and responsive to their wider health and circumstances [[1], [2], [3], [4], [5], [6], [7], [8], [9], [10]]. Newly qualified healthcare professionals, meanwhile, enter clinical practice with the foundational knowledge, skills, and perspectives shaped in part by their university education. Understanding how this educational preparation aligns with contemporary patient expectations may identify patient-relevant priorities for obesity education.

Patients living with obesity increasingly expect care that recognises obesity as a complex chronic disease and is individualised, evidence-based, and delivered through shared decision-making [6,10,11]. They value holistic discussions encompassing lifestyle, obesity medications and bariatric surgery, balanced information about treatment options, and outcomes extending beyond weight loss to include quality of life and psychological wellbeing [[1], [2], [3], [4], [5], [6], [7], [8], [9], [10]]. Despite these advances, patients continue to report stigma, judgement and unmet information needs that undermine trust and engagement with healthcare [5,12,13].

In parallel, research consistently demonstrates that healthcare professionals feel underprepared to deliver contemporary obesity care. University obesity education remains limited, with limited specific obesity-teaching on communication, obesity medications, behavioural interventions and weight stigma [[14], [15], [16], [17], [18]], while recent competency frameworks advocate broader education encompassing multidisciplinary management and patient-centred care [[19], [20], [21], [22]]. Despite these recommendations, healthcare professionals continue to report limited confidence, inadequate university preparation and a need for structured competency-based obesity education [23,24]. These findings describe educational preparation, however, rather than whether that preparation corresponds to the capabilities that patients themselves consider important.

Patient expectations and HCP educational preparation have largely been examined as separate areas of research. Considering them together provides a different perspective: areas of correspondence may identify aspects of obesity care recognised by both groups, while areas of divergence may identify patient-relevant aspects of care that are less consistently reflected in HCPs' reported educational preparation. Such comparison does not establish HCP competence or clinical performance, nor does it treat patient expectations as an objective measure of either. Rather, it examines the relationship between two complementary perspectives on what HCPs may need to bring to obesity-related clinical encounters.

The present study therefore undertook a secondary comparative qualitative analysis of two existing interview datasets collected from adults living with obesity and newly qualified HCPs. These datasets have previously been analysed separately: the patient study explored expectations and preferences regarding obesity consultations, while the HCP study examined experiences of university obesity education and preparedness for practice [10,23]. For the present study, the original interview transcripts were re-analysed in relation to a new comparative research question, rather than comparing the themes reported in the primary publications.

The aim was to characterise areas of perceived alignment and divergence between patient-reported expectations of HCPs and newly qualified HCPs' reported university educational preparation, and to identify patient-relevant priorities for future obesity education.

## 2 Methods

### 2.1 Study design

This was a secondary comparative qualitative analysis study involving two previously collected interview datasets. Full details of participant recruitment, sample characteristics, and original data collection procedures are reported in the primary publications and are summarised briefly below.

This study addressed a distinct comparative research question: *Does university education reported by newly qualified HCPs correspond to the capabilities that patients expect HCPs to bring to obesity care?* To address this question, the original interview transcripts from both cohorts were re-analysed comparatively. The analysis examined perceived correspondence between patient-reported expectations and HCP-reported educational preparation rather than objectively measured HCP knowledge or clinical performance. The two cohorts were recruited independently, interviewed using different semi-structured interview guides, and were not linked through shared clinical encounters. Accordingly, comparisons were made at the level of patterns across the two datasets rather than between individual patients and HCPs.

### 2.2 Original datasets and data collection

The secondary analysis included all transcripts from two previously reported interview datasets [10,23]: 20 adults living with obesity with current or previous experience of obesity treatment, and 21 newly qualified HCPs who had graduated within the preceding two years. Recruitment, eligibility, interview-guide development and data-collection procedures were undertaken as part of those primary studies and are reported in detail in the corresponding publications. No new participants, interviews or data were collected for the present study.

For context, both cohorts were purposively recruited for their respective primary studies and interviewed individually using semi-structured interview guides tailored to each study's own research question. Patient interviews were conducted between October and December 2024, exploring experiences of obesity care and expectations of clinical consultations. HCP interviews were conducted between November 2024 and May 2025, exploring university obesity education, clinical exposure and preparedness for practice. The interview guides are provided in Supplementary Files 1 and 2. Because the two guides addressed different research questions, the present analysis compared corresponding patterns across complementary perspectives rather than responses to identical questions.

### 2.3 Secondary comparative analysis

The patient and HCP datasets were initially coded separately, focusing on patient-reported expectations of HCPs and HCP-reported knowledge, confidence and educational preparation. This was done using MAXQDA (v24.7.0) software. Corresponding patterns were then compared across datasets to identify areas of perceived alignment and divergence, and refined through iterative discussion into higher-order comparative themes capturing the relationships between patient expectations and HCP-reported preparation while preserving the distinction between the two perspectives. Analysis was conducted independently by TYLK and FC, with themes reviewed and refined with the wider research team (CWLR, WAN).

### 2.4 Reflexivity and methodological rigour

TYLK conducted all original interviews and had prior experience conducting qualitative research in obesity. The wider research team included researchers and clinicians with backgrounds in medicine and dietetics, several of whom work predominantly in obesity care and research. These backgrounds provided relevant disciplinary knowledge but also had the potential to influence interpretation of the data. Reflexive discussion was therefore incorporated throughout the comparative analysis, with researchers examining assumptions arising from their clinical and research backgrounds and considering alternative interpretations of the data.

The comparative analysis maintained a clear distinction between the epistemic position of the two cohorts. Patient accounts were not used as objective measures of the knowledge or performance of the HCP cohort, and HCP self-reports were not interpreted as direct observations of clinical competence. Analytic discussions instead focused on whether patterns in HCP-reported educational preparation corresponded to the capabilities described as important within patient accounts. Quotations and candidate themes were repeatedly considered in the context of the complete transcripts to ensure that the comparative interpretation remained grounded in participants' accounts.

### 2.5 Sample adequacy

As a secondary analysis, using all available transcripts (20 patient, 21 HCP), no new sample-size determination or recruitment stopping criterion was applied; sampling and sample adequacy for each cohort were established during the respective primary studies and reported there [10,23]. Of particular relevance to the present comparative analysis, no substantially new patterns were identified in the HCP study after 19 interviews, after which two further interviews were conducted to ensure breadth across professional disciplines.

### 2.6 Ethical considerations

The original studies received approval from the University College Dublin Human Research Ethics Committee [Reference: HS-20-12-McGillicuddy; Reference: LS-24-69-Najim]. All participants provided written informed consent before participation. Identifying information was removed during transcription, and study data were handled in accordance with the approved ethical procedures.

Reporting of the present study was guided by the Standards for Reporting Qualitative Research (SRQR) [25], with the completed reporting checklist provided as Supplementary File 3.

## 3 Results

The analysis included 41 participants: 20 adults living with obesity and 21 newly qualified healthcare professionals (HCPs). The HCP cohort included participants from medicine (n = 16), physiotherapy (n = 2), occupational therapy (n = 1), dietetics (n = 1) and nursing (n = 1).

The comparative analysis generated four themes describing areas of perceived alignment and divergence between patient-reported expectations and HCP-reported university educational preparation. Across the datasets, patients and HCPs often identified similar aspects of obesity care as important, while differences emerged in the depth, application and integration of these areas within clinical care and educational preparation. The four themes were: (1) Beyond Recognising Obesity as Multifactorial; (2) Sensitivity Requires More Than Sensitive Language; (3) Clinical Orientation to Obesity Treatment and Care Pathways; and (4) Fragmented Education and the Expectation of Integrated Obesity Care Table 1.

**Table 1:** Overarching table of themes & analytic framework.

Theme	Area of broad alignment	Patient-reported expectations	HCP-reported university preparation	Comparative synthesis
**1. Beyond Recognising Obesity as Multifactorial**	Both cohorts recognised that obesity is multifactorial and influenced by multiple biological, psychological, behavioural and social factors.	Patients expected this understanding to reduce blame, move care beyond weight or BMI, account for their wider health and circumstances, and inform individualised management. Patients prioritised factors aside from weight alone in these discussions.	HCPs commonly recognised obesity as multifactorial, but some described remembering concepts rather than specifics, having “surface-level” knowledge, or finding it difficult to explain and apply the contributing factors. Some HCPs described difficulty moving beyond weight-based management in clinical practice.	Alignment in recognising multifactoriality did not consistently extend to its clinical application. Patients expected an understanding of complexity to shape clinical reasoning and individualised care, while HCPs reported variable preparation for applying multiple determinants to the individual patient.
**2. Sensitivity Requires More Than Sensitive Language**	Both cohorts recognised obesity as a sensitive and potentially stigmatising subject and considered sensitive communication important.	Patients understood sensitivity as more than polite or permission-based language. They expected HCPs to avoid assumptions and blame, recognise the heterogeneity of obesity, understand relevant circumstances and provide contextually appropriate advice.	Some HCPs recalled teaching about stigma, bias or permission-based approaches. However, opportunities to explore obesity-specific stigma or practise conversations beyond their initiation were inconsistent.	The divergence concerned the clinical understanding underpinning communication. Patients expected sensitivity to reflect obesity-specific knowledge and reasoning, whereas HCPs reported variable preparation for integrating communication principles with such understanding.
**3. Clinical Orientation to Obesity Treatment and Care Pathways**	Both datasets identified treatment knowledge and appropriate management as important.	Patients expected HCPs to recognise multiple treatment approaches, move beyond repetitive generic lifestyle advice, discuss relevant possibilities and facilitate access to further expertise when appropriate.	HCPs reported heterogenous and variable levels of knowledge and confidence regarding treatment escalation, obesity medications, surgery, referral pathways and what should follow unsuccessful initial management. Preparation also varied by profession and clinical exposure.	The main divergence concerned moving from recognising obesity to taking an appropriate next step. Patients expected constructive, treatment-oriented discussions, while HCPs reported heterogeneous preparation for connecting treatment knowledge with clinical decisions and onward care.
**4. Fragmented Education and the Expectation of Integrated Obesity Care**	Both cohorts described fragmentation in relation to obesity: patients reported fragmented care across conditions and specialties, while HCPs described obesity-related learning as fragmented across subjects, disease contexts and clinical settings.	Patients expected HCPs to recognise how obesity related to their wider health and to connect care across conditions, specialties and episodes of treatment. Some described care in which complications were addressed separately without obesity becoming a coherent focus.	HCPs described obesity-related learning as dispersed across subjects, disease contexts and clinical placements. They sometimes had substantial exposure to obesity-related complications without experience of obesity itself as the focus of care.	Corresponding forms of fragmentation were identified across the datasets. Patients experienced fragmented care, while HCPs described fragmented learning. HCPs' accounts did not consistently indicate that these separate areas of learning had been integrated into a coherent framework for obesity care.

*This table presents perceived correspondence between patient-reported expectations and HCP-reported university preparation. It does not compare matched patient–HCP encounters or represent an objective assessment of HCP knowledge, competence or clinical performance.

### 3.1 Theme 1. Beyond Recognising Obesity as multifactorial

Both cohorts recognised obesity as a multifactorial disease, creating apparent alignment at the level of principle. Patients described biological, psychological, behavioural and social influences, while HCPs similarly identified genetic, endocrine, medication-related, psychological, cultural and socioeconomic contributors. However, the cohorts diverged in what this understanding was expected to enable.

For patients, recognising multifactoriality was expected to shape how HCPs understood and cared for the individual: reducing behavioural attribution and blame, looking beyond weight or body mass index (BMI), considering how obesity related to wider health and quality of life, and tailoring management to individual circumstances. One participant identified the “anatomy and physiology of obesity” and, repeatedly, understanding “the why” as priorities for HCP education, explicitly connecting limited understanding with stigma and guilt.

One patient mentioned “*they might have known a lot about food. but they didn't know a lot about me, or why I was obese. and that it wasn't just a matter of saying to me, Okay, you're going to eat less, and these are the foods that you're going to eat. I had that knowledge. you know, but I couldn't control it if it makes any sense*”, highlighting the importance of individualisation. This quote: “*It's not just about self-control, you know or about knowledge about food. or how to eat healthily. But I think maybe people different people are obese for different reasons. and that is something that you know obesity management specialists should explore so that they can target the they can target their therapies appropriately*” further emphasises how patients see multifactoriality being operationalised within obesity care.

HCP transcripts revealed a distinction between knowing that obesity is multifactorial and being able to apply this understanding clinically. One participant described remembering “concepts more than specifics”: although “multifactorial” had been retained, discussing the individual factors was difficult. Others identified multiple contributors but struggled to explain obesity pathophysiology, while some characterised their knowledge as “very surface level.” HCP accounts provided less evidence that university education had translated this recognition into a framework for understanding the individual patient. Obesity was often approached through more readily taught and measurable constructs, including BMI, weight, associated risk and lifestyle modification. One respondent summarised: *“If a patient was ever noticed to be obese, it was always in the management plan of ‘lose weight’ … but not how to actually manage the obesity itself”.* In contrast, one patient stated that “*if healthcare professionals … are giving me advice. I want them to listen to me and to adapt their advice to the lifestyle*”. Patients expected multifactorial understanding to inform clinical reasoning by situating weight within a broader picture incorporating biological drivers, mental health, mobility, comorbidities and individual circumstances.

One patient illustrated this by saying: “*I think, what do I think is most important? This was for me the most important, like I said is kind of getting that wide cover across various disciplines, understanding that there's psychological elements, understanding that there is nutritional elements, that there's physical element,s that there's genetic elements, and bringing the patient along that journey of understanding that to allow them to make their choice as to what suits them best for weight management*.“

The divergence concerned the clinical operationalisation of multifactoriality. Patients expected understanding of obesity's complexity to support explanation, reduce blame and guide individualised care, while HCPs reported variable university preparation for incorporating multiple determinants into their understanding of the individual patient and the context of appropriate management.

### 3.2 Sensitivity Requires More Than Sensitive Language

Both cohorts recognised obesity as a sensitive and potentially stigmatising topic. Patients expected weight to be discussed without judgement or blame, with HCPs similarly recognised the importance of sensitivity, with some recalling teaching on stigma or permission-based approaches to initiating weight discussions. Comparison across the cohorts, however, showed differences in the depth of understanding underpinning these conversations.

For patients, sensitivity extended beyond language or interpersonal manner. It was reflected in whether the HCP recognised the heterogeneity of obesity, avoided assumptions about individual responsibility, considered circumstances relevant to the person's obesity, and provided advice or direction appropriate to that context. Patients therefore connected sensitive communication with obesity-specific understanding. A conversation could be conducted politely yet remain reductive if the underlying clinical reasoning continued to frame obesity primarily in terms of individual behaviour or weight. In this instance, the sensitivity became tokenistic and superficial, rather than truly sensitive and aligned with the understanding of the condition of obesity. Their accounts positioned sensitivity as an application of understanding within the clinical encounter.

One patient illustrated this by stating: “*people have been judged their whole life. They don't need their obesity specialist to be judging them as well. They need your help. And they need you to understand that there are times that they struggle with specific things. And how can you help me? You know my barriers, or you know if I can't overcome them. Can you make them a little bit smaller, so that my journey is a little bit easier?*“. Another echoed this, saying: “*So I think it comes a lot about that understanding of each other and the establishing of rapport and having someone who is empathetic. So they understand where you're coming from and what your perspective is*”.

HCP accounts suggested that university education did not consistently make this connection. Stigma was sometimes incorporated within broader teaching on bias or acknowledged principally as a barrier to treatment, with limited exploration of how obesity stigma might shape the clinical encounter. Even where participants recalled instruction on initiating weight discussions sensitively, some described little opportunity to practise or develop the conversation beyond its initiation. This occurred alongside educational experiences in which obesity was frequently encountered as a risk factor or comorbidity, with variable obesity-specific teaching. One HCP further stated: “*I will be comfortable [discussing obesity], but I will be scared that I will make the another person not comfortable*”.

One HCP described it clearly: “*I think that the level that I have, and that a lot of my peers would have is that we're very aware of the kind of general concepts of obesity that we're aware of the stigma and surrounding it, and that we're aware of again, the general concepts of management, but that maybe the specifics like how to actually have those discussions in practice, how to refer on or how to counsel patients about different options is maybe something that people at my level would not be as confident with*”.

The comparative divergence therefore concerned the clinical substance underpinning sensitive communication. Patients expected sensitivity to be informed by sufficient understanding of obesity to recognise complexity, avoid reductive assumptions and respond appropriately to the individual. HCPs reported exposure to stigma awareness and communication principles, but their preparation for integrating these principles with obesity-specific understanding varied.

### 3.3 Clinical orientation to Obesity Treatment and Care Pathways

Both datasets positioned treatment knowledge as important, while the comparative analysis highlighted the importance of applying this knowledge within a clinical encounter. Patients expected HCPs to recognise that obesity has multiple management approaches and, where appropriate, to discuss relevant possibilities or facilitate access to further expertise. Several patient accounts described frustration when obesity care stopped at generic lifestyle advice despite previous attempts at weight management, while others explicitly valued HCPs knowing what options existed and where patients could be referred for further care, for example, one patient wished that “*they know where to send people, and that they don't look just at your weight. For, like every problem. and that they know where to refer you to. That it's not just a lose weight is the answer to everything*”.

One patient stated: “*I think quite a lot of them [clinicians] don't know what the options are. They hear about things like there is medications coming down the roof.…I think that it'd be great if they go. Okay. Your options are: There are surgical options. There are medication options. There are options that are, you know, related to kind of diet, plans and nutrition, and all those kind of things as well, and just knowing what they are and who is available to take patients in those areas, I think would be great*”. Another said: “*They should be able to know what the surgical options are for you … what they are publicly and privately they should know what the medication options are for you. They should know where to refer you for your area*”.

HCP accounts showed variable university preparation across this treatment landscape. Some participants could identify broad treatment modalities or had developed greater knowledge through particular clinical exposures, while others described uncertainty regarding treatment escalation, obesity medications, surgical indications or referral pathways. In some accounts, awareness that particular treatments existed was accompanied by less confidence about when they might be considered, how they could be discussed with a patient, or what should follow unsuccessful initial management. These differences were also shaped by professional role, with participants varying in the obesity-related knowledge and management responsibilities they considered relevant to their scope of practice.

One HCP mentioned: “*I remember the concepts of and the different types of surgeries that are available. But I find it difficult to remember the specific indications and risks for each surgery*”. Another quote echoed this: “*I really wouldn't be confident, having an in-depth discussion with a patient about lifestyle measures for options for management of obesity*”. Another HCP mentioned: “*I actually don't try and talk to the patients about [semaglutide] … because I actually don't know current criteria for accessibility*”.

The comparison highlighted clinical orientation to obesity care: the capacity to connect treatment knowledge with the next steps required when obesity becomes relevant within an encounter. Patients expected HCPs to move from recognising obesity as clinically relevant to a constructive discussion of management, including what might appropriately be considered next and when further expertise was required. HCPs reported heterogeneous confidence in making these transitions, alongside variation in their obesity education and obesity-specific clinical exposure.

Across the datasets, generic diet and physical activity advice also emerged as a recurring feature. Patients described such advice as repetitive and insufficiently responsive to previous efforts or individual circumstances and, in some accounts, associated reductive lifestyle framing with blame and weight stigma. Some HCP accounts similarly showed greater familiarity with broad lifestyle recommendations than with treatment escalation or onward care. Importantly, encountering patients living with obesity in routine practice did not necessarily provide experience of addressing obesity itself as a clinical problem. Treatment knowledge, communication and pathway awareness emerged as interconnected aspects of the clinical preparedness required to address obesity within routine practice.

### 3.4 Fragmented Education and the Expectation of Integrated Obesity Care

Patients described obesity as a condition connecting multiple aspects of their health, while their experiences of healthcare could separate these into individual problems managed across different clinical contexts. Obesity-related complications could be addressed individually, with weight identified as a contributing factor, without obesity itself becoming a coherent focus of assessment, discussion or management. One participant described receiving care for diabetes, sleep apnoea, cardiac and other conditions across different specialists as “very splintered,” contrasting this experience with an expectation that HCPs recognise obesity as an “overall binding condition” across these aspects of health.

One patient stated: “*Everybody was treating the symptoms as they popped up, and I was running myself between pillar and post from specialist to specialist. I didn't perceive the holistic question either myself. Only, as I say with hindsight*”. Another patient stated: “*my experience was that I was treated very much to the series of fractured, a series of conditions, various related conditions relating to my pancreas and my joints, and … my heart, and in my sleep, and my lungs, and God knows where else, and various specialists, and all of it was very splintered. There was not a holistic viewpoint”*.

HCP accounts described a parallel fragmentation in how obesity was encountered during university education. Obesity-related content was distributed across subjects and clinical contexts, including diabetes, metabolic disease, surgery, cardiovascular risk and health promotion. Within these contexts, obesity could appear as a risk factor, comorbidity or contributor to another condition, with less emphasis on bringing these components together around obesity itself. One HCP, for example, recalled obesity being discussed alongside diabetes in endocrinology and within surgical teaching, while other coverage was remembered as limited in depth. One HCP described it as “*majority of the conditions that we would be learning about, they'd be like, oh, it can be caused by obesity! But that that was it. There was nothing more mentioned. It was just like a list of causes, and there was nothing much else*”, another said “*all of the teaching surrounding obesity was in relation to other conditions*”. This pattern extended into clinical exposure: some participants reported considerable experience managing obesity-related conditions or caring for people with obesity while having limited experience of obesity itself as the focus of care, to the extent that one HCP stated that “*I haven't witnessed any treatment for obesity at all. I don't think and it was always as a comorbidity*”. One participant explicitly described having “much more” exposure to obesity-related conditions and complications than to “actual obesity in and of itself”. Another said: “*If you see a patient, you deal with them in terms of what they're presenting with, but you might know, based on their weight and their height and their waist circumference, that they are classed as obese. But again, you can only treat what they're presenting with*”.

The comparative analysis therefore identified corresponding forms of fragmentation across the two datasets. Patients described fragmentation across conditions, specialties and episodes of care, while HCPs described encountering obesity across separate subjects, disease contexts and clinical placements. Patients expected HCPs to connect these elements by recognising the relevance of obesity across their wider health and care. HCP accounts indicated that university education did not consistently provide a similarly coherent framework for bringing obesity-related knowledge together around the person with obesity. The perceived divergence concerned the integration of obesity across clinical understanding: patients expected coherence across their care, while HCPs described educational preparation in which relevant knowledge was often encountered in separate contexts.

## 4 Discussion

This secondary comparative analysis identified both perceived alignment and divergence between what people living with obesity expected HCPs to bring to obesity-related clinical encounters and what newly qualified HCPs reported receiving through university education. Across both datasets, there was broad recognition of obesity as multifactorial disease, the importance of sensitive communication and the need for appropriate management. However, the comparison suggested that alignment at the level of these broad principles did not consistently extend to their clinical application. Patients expected understanding of obesity to inform individualised assessment, non-stigmatising communication, discussion of appropriate management options and integration of obesity within their wider health and care. HCPs described variable university preparation for applying obesity-related knowledge in these ways. A further comparative pattern concerned fragmentation: patients described obesity care distributed across conditions and specialties, while HCPs described obesity education distributed across subjects, disease contexts and clinical placements.

Existing literature has frequently characterised shortcomings in obesity education in terms of limited curricular time, knowledge or confidence [14,15,17,24,26,27]. The present comparison adds a patient-facing dimension to this literature. Patient expectations frequently concerned what obesity-specific understanding enabled an HCP to do: interpret obesity within individual circumstances, communicate without reductive assumptions, discuss appropriate management possibilities and recognise when further expertise was required. HCP accounts suggested that relevant concepts were encountered during university education, but that opportunities to connect these concepts to clinical decision-making and conversations about obesity were variable. Furthermore, similar literature has documented that many clinicians tend to avoid discussing obesity due to a lack of confidence in their ability to do so without negatively impacting the clinician-patient relationship [28], with some doctors report feeling unskilled to do so or signpost appropriately [29], and medical students deeming their training in obesity counselling to be inadequate [30,31]. This distinction between curricular exposure and clinical application may therefore be important when considering how university obesity competencies are defined and taught.

The findings also raise questions about how obesity education is organised within university curricula. Obesity intersects with multiple organ systems, clinical disciplines and professional roles [32,33], making some distribution of relevant teaching across curricula inevitable. The educational challenge may therefore be less merely the presence of obesity, than ensuring that learning encountered across different contexts is connected into a coherent understanding of obesity and its management. This distinction is relevant to competency frameworks advocating multidisciplinary and person-centred obesity care [[19], [20], [21],^34^]. Although the present study cannot establish a causal relationship between the fragmentation described in education and patients' experiences of fragmented care, the parallel identifies coherence as a relevant consideration for both education and clinical practice. Although there is literature documenting both the recognition of greater obesity education in clinical training and the challenges of a crowded curriculum [14], a potential middle-ground is greater integration of what is already covered rather than a new allocation of dedicated training time. Greater curricular integration could help learners recognise obesity across clinical contexts while understanding how their own professional role connects with the contributions of other HCPs and specialist services.

The comparison further suggests that person-centred obesity care should not be conceptualised as a communication competency separate from clinical knowledge. In patient accounts, sensitivity was closely connected to whether HCPs understood obesity sufficiently to avoid simplistic behavioural explanations, contextualise the patient with an awareness of weight stigma, and respond to individual circumstances. In previous studies, patients have reported detailed accounts on how obesity treatment have affected them, and in-depth narratives on the implications both having obesity, and obesity treatments have on their lives [8,11,35], which suggests adequate knowledge on the obesity landscape is useful to facilitating engagement in obesity discussions. Similarly, expectations around treatment did not necessarily require every HCP to provide specialist obesity management; they included recognising legitimate treatment approaches, discussing options appropriate to professional scope and facilitating onward care when needed. University preparation may therefore benefit from integrating obesity-specific knowledge, communication and pathway awareness within clinically situated learning rather than treating these as independent competencies. It has been recommended that targeting training towards early in healthcare education may also be particularly helpful, especially in regards to weight stigma [36].

These findings should be interpreted as a comparison of perspectives rather than a direct assessment of educational effectiveness. The value of bringing the datasets together lies in identifying whether areas emphasised by patients are visible within newly qualified HCPs' accounts of their educational preparation. Neither dataset alone could address this relationship: the patient dataset identified what participants considered important in obesity consultations, while the HCP dataset identified how graduates experienced their university preparation. Their comparison highlights patient-relevant areas for educational consideration without positioning patient expectations as an objective standard against which HCP competence can be measured.

### 4.1 Strengths

By bringing together the perspectives of people living with obesity and newly qualified HCPs, it moves beyond single-stakeholder accounts and provides a novel patient-informed perspective on obesity education. The inclusion of 41 participants across two complementary datasets provided substantial qualitative material, with in-depth interviews generating rich accounts of both patient expectations and graduates’ experiences of educational preparation. Rather than comparing previously published findings, the analysis returned to the complete original transcripts, enabling systematic examination of a new comparative question while preserving the distinct perspectives of each cohort. This approach extends existing literature describing patient experiences and HCP education separately by examining where these perspectives correspond and diverge.

### 4.2 Limitations

As a secondary analysis, it compared two independently recruited cohorts originally interviewed for different research questions using cohort-specific interview guides; participants were not linked through shared clinical encounters. Findings therefore represent perceived correspondence between patient expectations and HCP-reported educational preparation rather than direct measures of educational effectiveness, knowledge or clinical competence. The HCP sample was predominantly medical graduates, limiting profession-specific interpretation, while patients had current or previous experience of obesity treatment and may not represent people receiving obesity care in primary or other healthcare settings. Finally, because no new data were collected, the original interviews were not designed specifically for the comparative research question. However, returning to the complete original transcripts enabled a distinct question to be examined that neither primary study could address independently, providing insight into how patient-described expectations of obesity care correspond with newly qualified HCPs’ reported educational preparation.

### 4.3 Implications and key recommendations

The findings suggest that strengthening university obesity education may require greater emphasis on the application and integration of knowledge, rather than simply increasing exposure to individual obesity-related topics. Across the comparative analysis, patient expectations frequently concerned how HCPs used their understanding of obesity within clinical encounters, while HCPs described variable preparation for translating relevant knowledge into communication, management discussions and onward care. University curricula should therefore consider how obesity-related learning encountered across different subjects and clinical settings can be connected into a coherent foundation for practice, while recognising differences in professional role and scope. The findings also support incorporating patient perspectives alongside professional competency frameworks when developing and evaluating obesity curricula.

## 5 Conclusions

This study identified both perceived alignment and divergence between what people living with obesity expect from HCPs and the university educational preparation reported by newly qualified HCPs. The findings suggest that university obesity education may benefit from greater emphasis on connecting foundational knowledge with its application in person-centred communication and navigating care and discussion when obesity is involved. Incorporating patient perspectives alongside evidence-based professional competencies may help ensure obesity education remains responsive to patient needs while preparing graduates for care appropriate to their professional scope.

## 6 Key takeaways

1. Obesity education should prioritise the clinical application of knowledge along with greater exposure to obesity-related content.
2. Obesity-specific knowledge, sensitive communication and weight-stigma awareness should be taught as interconnected clinical competencies rather than separate topics.
3. Education should focus on translating obesity knowledge into appropriate treatment discussions, recognition of professional scope and navigation of onward care.

## Consent for publication

All authors and participants consent for this study to be published.

## Availability of data and materials

The data generated and analysed during the current study are available from the corresponding author on reasonable request.

## Authors’ contributions

LK, CLR, and WA conceived and designed the study. LK recruited participants and conducted the semi-structured interviews. LK and FC performed the data analysis and drafted the manuscript. CLR, WA, and FC critically revised the document and approved the final version of the manuscript. The corresponding author attests that all listed authors meet authorship criteria and that no others meeting the criteria have been omitted.

## Declaration of generative AI and AI-assisted technologies in the manuscript preparation process

During the preparation of this work, the authors used ChatGPT (OpenAI, GPT-5.6 Sol) for the sole reason of grammar correction. The authors reviewed and edited the output as needed and take full responsibility for the content of the published article. No generative AI or AI-assisted technologies were used in the study design, data collection, data analysis, interpretation of results, or the generation of scientific conclusions.

## Funding

This research was funded by the European Commission’s Erasmus Mundus Design Measure grant number 101128158. This manuscript is part of the Stratification of Obesity Phenotypes to Optimize Future Obesity Therapy (SOPHIA) project (www.imisophia.eu). SOPHIA has received funding from the Innovative Medicines Initiative 2 Joint Undertaking under grant agreement No. 875534. This Joint Undertaking support from the European Union’s Horizon 2020 research and innovation program and EFPIA and T1D Exchange, JDRF, and Obesity Action Coalition. It should be made clear in the text and layout that the communication reflects the author's view and that neither IMI nor the European Union, EFPIA, or any Associated Partners are responsible for any use that may be made of the information contained therein.

## Conflict of interest statement

CLR reports grants from the Irish Research Council, Science Foundation Ireland, Anabio, and the Health Research Board. He serves on advisory boards of Novo Nordisk, Herbalife, GI Dynamics, Eli Lilly, Johnson & Johnson, Glia, Keyron, and Boehringer Ingelheim. CLR is a member of the Irish Society for Nutrition and Metabolism, outside the area of work commented on here. He served as the Chief Medical Officer and Director of the Medical Device Division of Keyron in 2021. Both were unremunerated positions. No patients have been included in any of Keyron's studies, and the company is not publicly traded. CLR was gifted stock in Keyron in September 2021 and divested all holdings the same month. He continues to provide scientific advice to Keyron without remuneration. W.A.-N. reports honoraria for presentations from Novo Nordisk and Boehringer Ingelheim. W.A.-N. provides dietetic services to ProHealth365 Physiotherapy & Nutrition and BeyondBMI, private obesity clinics.

All other authors declare no conflicts of interest.
