# Supplementary material for: Patient expectations and healthcare professional education in obesity care: A comparative qualitative study
Source: Obes Pillars. 2026 Sep 28;20:100337. doi: 10.1016/j.obpill.2026.100337 (PMC13653468; doi:10.1016/j.obpill.2026.100337)
Supplement: Supplementary file 1 [file mmc1.docx]

**Supplementary File 1.**

**For Patients with Obesity – Interview Guide**

This set of structured interview questions is aimed at gathering insights from adult patients who have a clinical diagnosis of obesity and have undergone various forms of treatment or intervention, such as lifestyle counselling, dietary management, medication, surgery, or behavioural therapy. The purpose of these interviews is to explore patients' experiences with obesity diagnosis, their interactions with healthcare providers, the effectiveness of different treatments, and the barriers they face in managing their weight.

**Inclusion criteria**

Adults patients aged 18 or older with the condition of obesity (BMI ≥ 27) with a complication of obesity who are currently receiving or in the past, have received some form of treatment or intervention for obesity, such as lifestyle counselling, dietary management, medication, surgery, or behavioural therapy.

**Introduction Script**
Obesity is a significant global health issue, yet there's a notable lack of standardized training for healthcare professionals. This interview will be used to develop a competency framework to address training gaps, improve obesity management, and enhance patient outcomes. Our goal is to establish a European standard pathway that integrates various disciplines, including behavioural, psychological, pharmacological, and surgical approaches. During this interview, we'll discuss your experience with obesity, including diagnosis, treatments you've tried, and your interactions with healthcare providers. We also want to understand the challenges you've faced in managing your weight and how we can better support you. Your feedback will help us improve the training of healthcare professionals and enhance the care provided to individuals with obesity. May I ask you a few questions about your experience?

1. **Experience with Obesity Diagnosis**
   - Can you tell me about how your weight has changed over time?
   - How you ever been diagnosed with obesity? If yes:
   - Can you share your experience of being diagnosed?
   - How did the diagnosis process make you feel?
   - Were you given sufficient information and support at the time of your diagnosis?
2. **Treatment Experience**

- What types of treatments or interventions have you tried for managing obesity that were prescribed or supervised by a healthcare provider specializing in obesity care?
- How effective were these treatments in managing your weight and overall health?
- Did you feel that your treatment options were explained clearly to you?

3. **Interaction with Healthcare Providers**

- How would you describe your interactions with healthcare providers regarding obesity management?
- Did you feel listened to and understood by your healthcare providers?
- Were your personal goals and preferences taken into account during your treatment?

4. **Barriers to Effective Treatment**

- What challenges have you faced in trying to manage your weight or follow treatment plans?
- Were there any barriers (e.g., financial, psychological, social) that made it difficult for you to access or adhere to treatment?
- How could healthcare providers or the healthcare system better support you in overcoming these barriers?

5. **Expectations from Healthcare Professionals**

- What qualities do you think are most important for healthcare professionals who specialize in obesity management?
- How important is it for healthcare professionals to have advanced training in obesity management?
- What specific knowledge or skills do you believe healthcare providers should have to better support patients with obesity?

6. **Feedback on Obesity Management Education**

- Do you think that more advanced training for healthcare professionals, like an MSc program, would improve the quality of care for patients with obesity?
- What topics or areas of knowledge do you believe should be included in an obesity management training program for healthcare professionals?
- How do you think such a program could improve the overall experience and outcomes for patients like you?

7. **Overall Satisfaction and Suggestions**

- How satisfied are you with the care and support you have received for managing obesity?
- If you could change one thing about the way obesity is treated in the healthcare system, what would it be?
- Do you have any additional suggestions or thoughts on how healthcare professionals can better support patients with obesity?

**Supplement 2.**

**Structured Interview Questions: Baseline Knowledge and Perspectives on Obesity Management for Newly Qualified Healthcare Professionals**

This set of structured interview questions aims to explore the experiences, knowledge, and expectations of newly-qualified, licensed healthcare professionals (e.g., medical doctor, nurse, dietitian, physiotherapist, psychologist, or other allied health professionals) currently practicing in a clinical setting. By capturing data from this cohort, we can accurately benchmark their educational exposure, knowledge, and competencies, as these newly-qualified professionals are recent graduates who can still recall their university training. We aim to identify and benchmark the level of knowledge, self-reported competency and exposure to obesity management from their university course.

**Inclusion criteria:**

Newly-qualified, licensed healthcare professionals who have less than 2 years work experience in a clinical setting.

**Introduction Script:**
Obesity is a significant global health issue, yet there's a notable lack of standardized training for healthcare professionals. This interview will be used to develop a competency framework to address training gaps, improve obesity management, and enhance patient outcomes. Our goal is to establish a European standard pathway that integrates various disciplines, including behavioural, psychological, pharmacological, and surgical approaches. Your experience as a newly qualified healthcare professional is crucial for understanding current educational exposure and competencies. May I please ask you a few questions regarding your experience with obesity medicine?

1. **Current Knowledge of Obesity Management**

- Can you describe what you currently know about obesity and its management?
- What do you believe are the primary causes of obesity?
- What treatment options or strategies are you familiar with for managing obesity?
- How would you describe the role of lifestyle changes, such as diet and exercise, in the management of obesity?
- How would you describe the role of pharmacotherapy in the management of obesity?
- How would you describe the role of surgery in the management of obesity?

2. **Previous Academic Exposure**

- Was obesity and obesity-related topics part of your university degree curriculum? If yes, what topics related to obesity were covered in your previous degrees or courses?
- How in-depth were the discussions or teachings on obesity management in your prior education? How were these delivered? (e.g. Lectures, on placement, workshops etc.)
- What key concepts or knowledge about obesity did you gain from your previous education?
- Was obesity taught as its own separate discipline or in dedicated lectures or was it embedded within other subjects/courses?
- Were there any projects, research, or papers you worked on that were related to obesity management? Can you describe them?

3. **Clinical or Practical Experience**

- Have you had any clinical experience or hands-on practice related to obesity management? If yes, can you elaborate on that experience?
- Have you ever observed or been involved in the treatment of patients with obesity? What was your role?
- How do you think practical experience has influenced your understanding of obesity management?

4. **Perception of Current Knowledge and Gaps**

- How confident are you in your current knowledge and understanding of obesity management?
- Are there areas of obesity management that you feel less confident or knowledgeable about?
- What do you perceive as the main challenges in managing obesity effectively?

5. **Importance of Obesity Management**

- In your opinion, how important is it for healthcare professionals to have specialized training in obesity management?
- How do you think improving knowledge in obesity management can impact patient care and outcomes?
- What role do you think healthcare professionals should play in preventing and managing obesity at a population level?

**Supplement 3. Standards for Reporting Qualitative Research (SRQR) Checklist**

|  | **Description in this study** | **Location** |
| --- | --- | --- |
| **Title & Abstract** | | |
| Title | The title identifies the topic as patient expectations and healthcare professional education in obesity care and explicitly identifies the work as a comparative qualitative study. | Title |
| Abstract | The structured abstract summarises the background, secondary comparative qualitative methods, the two cohorts (20 adults living with obesity and 21 newly qualified HCPs), the principal comparative findings, and the conclusion concerning integration and clinical application of obesity education. | Abstract |
| **Introduction** | | |
| Problem Formulation | The introduction describes changing obesity science and care, patient expectations for evidence-informed and individualised care, persistent stigma and unmet information needs, and evidence that HCPs remain underprepared. It identifies the knowledge gap that patient expectations and HCP educational preparation have largely been examined separately. | Introduction |
| Purpose or research question | The study aims to characterise perceived alignment and divergence between patient-reported expectations of HCPs and newly qualified HCPs’ reported undergraduate educational preparation for obesity care, and to identify patient-relevant priorities for future obesity education. The comparative research question asks whether reported university education corresponds to the capabilities patients expect HCPs to bring to obesity care. | Introduction; Methods - Study design |
| **Methods** | | |
| Qualitative approach | A secondary comparative qualitative analysis of two existing interview datasets. Original transcripts were re-analysed using thematic coding and cross-dataset comparison to identify areas of perceived alignment and divergence and develop higher-order comparative themes. | Methods - Study design; Secondary comparative analysis |
| Researcher characteristics and reflexivity | TYLK conducted all original interviews and had prior qualitative research experience in obesity. The wider team included researchers and clinicians from medicine and dietetics, working predominantly in obesity care and research. The manuscript acknowledges that these backgrounds could influence interpretation and describes reflexive discussion, examination of assumptions, and consideration of alternative interpretations. | Methods - Reflexivity and methodological rigour |
| Context | The secondary analysis used two independently recruited cohorts from previously reported studies. Patient interviews were conducted from October to December 2024 and explored experiences and expectations of obesity care; HCP interviews were conducted from November 2024 to May 2025 and explored undergraduate obesity education, clinical exposure and preparedness for practice. | Methods - Original datasets and data collection |
| Sampling strategy | Both cohorts were purposively recruited in their respective primary studies. The secondary analysis included all available transcripts: 20 adults living with obesity with current or previous obesity-treatment experience and 21 newly qualified HCPs who had graduated within the preceding two years. No new sample-size determination or stopping criterion was applied; in the HCP study no substantially new patterns were identified after 19 interviews and two further interviews were conducted for disciplinary breadth. | Methods - Original datasets and data collection; Sample adequacy |
| Ethical issues pertaining to human subjects | The original studies received University College Dublin Human Research Ethics Committee approval (HS-20-12-McGillicuddy and LS-24-69-Najim). All participants provided written informed consent. Identifying information was removed during transcription and data were handled in accordance with approved ethical procedures. | Methods - Ethical considerations |
| Data collection methods | The source datasets comprised individual semi-structured interviews conducted for the two primary studies. The manuscript provides the interview periods and the focus of each interview set and states that no new participants, interviews or data were collected for the present secondary analysis. Full recruitment and original data-collection procedures are reported in the primary publications. | Methods - Original datasets and data collection |
| Data collection instruments and technologies | Cohort-specific semi-structured interview guides were used, tailored to each primary study’s research question. Interviews were conducted via Zoom, recorded, transcribed and anonymized. The HCP study used a piloted interview guide and recorded field notes following interviews; the patient study used an open-ended interview guide developed specifically for the study. Full details of the instruments and data-collection technologies are reported in the respective primary publications, and the interview guides are provided in Supplementary Files 1 and 2. | Methods - Original datasets and data collection; Supplementary Files 1 and 2 |
| Units of study | The analysis included 41 participants across two cohorts: (1) 20 adults (≥18 years) living with obesity (BMI ≥27 kg/m² with ≥1 obesity-related complication) who had prior or ongoing obesity treatment; and (2) 21 newly qualified HCPs who had graduated from medical, nursing, or allied health programmes within the previous two years and were currently working in clinical practice. | Methods - Original datasets and data collection; Results |
| Data processing | Interviews were audio-recorded and transcribed verbatim, with identifying information removed during transcription. Transcripts from both cohorts were organised and coded using MAXQDA (v24.7.0). For the present secondary analysis, the complete original transcripts were re-examined and the patient and HCP datasets were initially coded separately before corresponding patterns were compared across datasets. Details on data processing for each cohort has been previously published. | Methods - Secondary comparative analysis; Ethical considerations |
| Data analysis | Patient and HCP datasets were initially coded separately around patient expectations and HCP knowledge, confidence and educational preparation. Corresponding patterns were then compared to identify perceived alignment and divergence and iteratively refined into higher-order comparative themes while preserving the distinction between perspectives. TYLK and FC analysed independently, with themes reviewed and refined with CWLR and WAN. | Methods - Secondary comparative analysis |
| Techniques to enhance trustworthiness | The analysis maintained a distinction between the epistemic positions of the two cohorts; patient accounts were not treated as objective measures of HCP performance and HCP self-reports were not treated as direct observations of competence. Reflexive discussion, consideration of alternative interpretations, team review, and repeated checking of quotations and candidate themes against complete transcripts were used to keep interpretations grounded in participants’ accounts. | Methods - Reflexivity and methodological rigour; Sample adequacy |
| **Results** | | |
| Synthesis and interpretation | The comparative analysis generated four themes: (1) Beyond Recognising Obesity as Multifactorial; (2) Sensitivity Requires More Than Sensitive Language; (3) Clinical Orientation to Obesity Treatment and Care Pathways; and (4) Fragmented Education and the Expectation of Integrated Obesity Care. Across themes, broad alignment in principles often diverged in clinical application and integration. | Results |
| Links to empirical data | Analytic findings are supported throughout the results by direct quotations from both patient and HCP interviews, including quotations concerning individualisation, stigma and empathy, treatment options and referral, and fragmented care and education. Table 1 also summarises the cross-dataset analytic framework. | Results |
| **Discussion** | | |
| Integration with prior work, implications, transferability, and contribution(s) to the field | The discussion situates the findings within literature identifying insufficient obesity education and calls for strengthened, competency-based training, including greater emphasis on person-centred care. While existing competency frameworks describe the knowledge and capabilities HCPs should develop, this study adds a patient-informed perspective on how these competencies need to translate into clinical practice. By comparing patient expectations with newly qualified HCPs’ reported educational preparation, the findings help clarify what person-centred obesity care may require from an educational perspective, including the integration and application of obesity-specific knowledge, sensitive communication, treatment options and care-pathway awareness. The study therefore contributes patient-relevant priorities that may inform the development and refinement of obesity curricula alongside existing professional competency frameworks. The scope of inference remains a comparison of perspectives rather than a direct assessment of educational effectiveness. | Discussion; Implications and key recommendations; Conclusions |
| Limitations | The limitations note that the study is a secondary analysis of independently recruited cohorts, with no shared clinical encounters; that the HCP sample was predominantly medical graduates; that patient participants had current or previous obesity-treatment experience and may not represent other settings; and that the original interviews were not designed specifically for the comparative research question. | Limitations |
| **Other** | | |
| Conflicts of interest | CLR reports grants from the Irish Research Council, Science Foundation Ireland, Anabio, and the Health Research Board. He serves on advisory boards of Novo Nordisk, Herbalife, GI Dynamics, Eli Lilly, Johnson & Johnson, Glia, Keyron, and Boehringer Ingelheim. CLR is a member of the Irish Society for Nutrition and Metabolism, outside the area of work commented on here. He served as the Chief Medical Officer and Director of the Medical Device Division of Keyron in 2021. Both were unremunerated positions. No patients have been included in any of Keyron’s studies, and the company is not publicly traded. CLR was gifted stock in Keyron in September 2021 and divested all holdings the same month. He continues to provide scientific advice to Keyron without remuneration. W.A.-N. reports honoraria for presentations from Novo Nordisk and Boehringer Ingelheim. W.A.-N. provides dietetic services to ProHealth365 Physiotherapy & Nutrition and BeyondBMI, private obesity clinics. All other authors declare no conflicts of interest. | Conflict of Interest Declaration |
| Funding | The research was funded by the European Commission Erasmus Mundus Design Measure grant 101128158 and forms part of the SOPHIA project, supported through IMI2 grant 875534 with European Union Horizon 2020, EFPIA and associated partner support. The statement notes that the communication reflects the authors’ view and that the named funders/partners are not responsible for use of the information. | Funding |
